# Expression of Melatonin and Dopamine D_3_ Receptor Heteromers in Eye Ciliary Body Epithelial Cells and Negative Correlation with Ocular Hypertension

**DOI:** 10.3390/cells9010152

**Published:** 2020-01-08

**Authors:** Irene Reyes-Resina, Hanan Awad Alkozi, Anna del Ser-Badia, Juan Sánchez-Naves, Jaume Lillo, Jasmina Jiménez, Jesús Pintor, Gemma Navarro, Rafael Franco

**Affiliations:** 1 Department of Biochemistry and Molecular Biomedicine, School of Biology, Universitat de Barcelona, 08028 Barcelona, Spain; lillojaume@gmail.com; 2 Neuroplasticity Research Group, Leibniz Institute for Neurobiology, 39118 Magdeburg, Germany; 3 Centro de Investigación en Red, Enfermedades Neurodegenerativas, CiberNed, Instituto de Salud Carlos III, 28029 Madrid, Spain; delserbadia@gmail.com (A.d.S.-B.); jasminajc@gmail.com (J.J.); 4 Department of Biochemistry and Molecular Biology, Faculty of Optics and Optometry, University Complutense of Madrid, 28037 Madrid, Spain; hanan-q1@live.com (H.A.A.); jpintor@ucm.es (J.P.); 5 Department de Bioquímica i Biologia Molecular, Institut de Neurociències, Universitat Autònoma de Barcelona, Cerdanyola del Vallès, 08193 Barcelona, Spain; 6 Department of Ophthalmology, Balearic Islands Institute of Ophthalmology, 07013 Palma de Mallorca, Mallorca, Spain; juansanchez.naves@gmail.com; 7 Department of Biochemistry and Physiology, School of Pharmacy and Food Sciences, Universitat de Barcelona, 08027 Barcelona, Spain; 8 School of Chemistry, Universitat de Barcelona, 08028 Barcelona, Spain

**Keywords:** circadian rhythm, glaucoma, G-protein-coupled receptor GPCR heteromer, melatonin MT_1_ and MT_2_ receptors, G protein coupling, mitogen-activated protein kinase pathway (MAPK), label-free dynamic mass redistribution (DMR), retina, human 59HCE cells

## Abstract

Background: Experiments in the late nineties showed an inverse relationship in the eye levels of melatonin and dopamine, thereby constituting an example of eye parameters that are prone to circadian variations. The underlying mechanisms are not known but these relevant molecules act via specific cell surface dopamine and melatonin receptors. This study investigated whether these receptors formed heteromers whose function impact on eye physiology. We performed biophysical assays to identify interactions in heterologous systems. Particular heteromer functionality was detected using Gi coupling, MAPK activation, and label-free assays. The expression of the heteroreceptor complexes was assessed using proximity ligation assays in cells producing the aqueous humor and human eye samples. Dopamine D_3_ receptors (D_3_Rs) were identified in eye ciliary body epithelial cells. We discovered heteromers formed by D_3_R and either MT_1_ (MT_1_R) or MT_2_ (MT_2_R) melatonin receptors. Heteromerization led to the blockade of D_3_R-Gi coupling and regulation of signaling to the MAPK pathway. Heteromer expression was negatively correlated with intraocular hypertension. Conclusions: Heteromers likely mediate melatonin and dopamine actions in structures regulating intraocular pressure. Significant expression of D_3_R–MT_1_R and D_3_R–MT_1_R was associated with normotensive conditions, whereas expression diminished in a cell model of hypertension. A clear trend of expression reduction was observed in samples from glaucoma cases. The trend was marked but no statistical analysis was possible as the number of available eyes was 2.

## 1. Introduction

The ciliary body, hidden behind the iris, is a complex, highly specialized tissue with a surface containing a series of radially arranged structures known as ciliary processes [1], which are densely vascularized and covered by highly active secretory cells to comply with the function of ciliary processes, i.e., aqueous humor production [2].

The aqueous humor is a clear transparent fluid which bathes the anterior and posterior chambers of the eye [3,4,5]. It participates in numerous functions such as providing nutrients to the cornea, lens, and trabecular meshwork. More importantly, it also contributes to keeping the force that supports intraocular tissue, thereby giving the eye a certain intraocular pressure (IOP) [6]. IOP is maintained within normal values through a dynamic balance between its constant production by the ciliary processes and its drainage. A lack of balance in the aqueous humor dynamics, usually leading to an increment of IOP, is a significant risk factor in specific ocular pathologies such as glaucoma, the second leading cause of blindness worldwide [7].

Several factors contribute to the homeostasis of IOP, among others, the episcleral vein pressure, the ratio between production and drainage of aqueous humor, the influence of hormones, the innervation by cranial nerves V and VII, and the circadian rhythm. Evidence showed that IOP follows variations throughout the day, being maximal in the early morning and reaching its minimal levels during the night [8]. These fluctuations are small, between 3–5 mm Hg, in healthy subjects. However, they are significantly higher in the glaucomatous eye [9]. The primary modulator of eye circadian rhythms seems to be melatonin.

Melatonin, an indoleamine that participates in the control of circadian rhythms, is a neurohormone first identified in the pineal gland. However, a decade after its discovery by the dermatologist Aaron Lerner and colleagues [10], it gained particular interest because of its multifunctional role. Apart from being synthesized out of the pineal gland, melatonin is also considered an antioxidant and it contributes to minimize the circadian disruption resulting from jet lag or in shift workers [11,12]. Melatonin is synthesized by the retina [13] but, in subsequent studies, other sources were detected, namely, the iris, the ciliary body [14], the crystalline lens [15,16], the Harderian gland [17] and the lacrimal gland [18]. It seems that melatonin can function directly as a free radical scavenger, but it mainly acts via specific cell surface melatonin receptors. 

In mammals, two melatonin receptors, MT_1_ (MT_1_R) and MT_2_ (MT_2_R) have been cloned and characterized [19,20]. These receptors are present in numerous ocular structures; for instance, they are expressed in the cornea, and among other functions, they accelerate corneal wound healing [21,22]. They mediate regulation of circadian rhythms and neuroendocrine processes in the retina and in the non-pigmented ciliary body epithelium, which mediates IOP control (see References [23,24] and references therein).

Melatonin receptors are members of the 7-transmembrane G protein-coupled receptor family (GPCRs). Both MT_1_ and MT_2_ receptors belong to the class A of rhodopsin-like GPCRs (https://gpcrdb.org/) and contain seven hydrophobic transmembrane helices. Melatonin receptors have a well-established canonical signaling pathway, where the stimulation of MT_1_R and MT_2_R results in adenylate cyclase (AC) and protein kinase A inactivation by Gi-coupling [25,26,27]. However, it has been reported that, depending on the tissue or the species studied, melatonin receptors could couple to other G proteins to, for instance, activate phospholipase C-α (PLCα) [28] or inhibit guanylate cyclase (GC) [29]. Interestingly, studies show that the activation of melatonin receptors in the ciliary processes would lead to an increase in cAMP levels [30]. Other possibilities include the influence derived from interactions between these receptors and other receptors or other non-receptor proteins [31]. In this sense, direct interactions between melatonin receptors and GPR50 have been reported. This orphan GPCR may directly interact with the two melatonin receptors, although the interaction does not alter MT_2_R but MT_1_R coupling to Gi [32]. The MT_2_R also interacts directly with 5-hydroxytryptamine 5HT_2c_ receptors, the heteromer leading to biased agonism for the melatonin receptor ligand, agomelatine [33]. Although little is known about melatonin receptor heteromerization in the eye, a recent report has demonstrated the heteromerization of melatonin and α_1_-adrenergic receptors that alters canonical coupling as heteromerization impedes coupling to the cognate G proteins, Gi and Gq, respectively [26,34].

Dopamine is present in eye structures, and the extracellular level of this neurotransmitter regulates the synthesis of melatonin (in the eye) via dopamine receptors and cAMP as a second messenger [35,36,37,38]. The first evidence of an inverse correlation between dopamine and melatonin was found in the pigeon eye. Microdialysis assays found that dopamine concentration decreased when melatonin levels increased. In addition, melatonin injection suppressed the release of dopamine. Subsequent studies in the same laboratory confirmed that melatonin/dopamine interactions were complex but relevant for circadian rhythms in the eye [39,40]. However, the mechanisms underlying dopamine–melatonin relationships in the eye have not yet been elucidated.

Dopamine receptors are grouped into two subfamilies: D_1_-like, which includes D_1_ and D_5_, and D_2_-like, which includes D_2_, D_3_, and D_4_ [26]. Dopamine receptor expression has been addressed directly in the retina. Dopamine D_4_ receptors were detected in the vertebrate retina [41] and D_2_ and D_3_ receptors were identified in the human retina by targeted mass spectrometry and positron emission tomography [42]. Indirect evidence based on pharmacological assays and on responses in D_3_ receptor (D_3_R) KO mice showed that D_3_R activation leads to IOP reduction [43,44,45]. In preliminary experiments, we have detected the D_3_ receptor in human ciliary body epithelial cells. The present work aimed to address the potential interaction between dopamine D_3_ and melatonin receptors, along with the functional consequences of these interactions. The expressions of D_3_R–MT_1_R and D_3_R–MT_2_R heteroreceptor complexes were quantified in a ciliary body-based cell model of elevated IOP, and they were assessed in samples from human normotensive and hypertensive eyes. The results proved heteromerization and provided evidence of a negative correlation between ocular hypertension and heteromer expression.

## 2. Materials and Methods

This work adhered to the guidelines detailed in Reference [46] and to the Helsinki declaration regarding the ethics of working with human samples. The studies were designed to generate groups of equal size, using randomization and blinded analysis. Immuno-based assays were conducted in line with guidelines detailed in Alexander et al., 2018 [47].

### 2.1. Drugs

(RS)-*trans*-7-Hydroxy-2-[*N*-propyl-*N*-(3′-iodo-2′-propenyl)amino]tetralin (7-OH-PIPAT) maleate (mixed D_2_R/D_3_R agonist), 3,5-Dichloro-*N*-[[(2*S*)-1-ethyl-2-pyrrolidinyl]methyl]-2-hydroxy-6-methoxybenzamide (raclopride: D_3_R antagonist), *N*-Acetyl-5-methoxytryptamine (melatonin), N-qcetyl-2-benzyltryptamine (luzindole: non-selective MTR antagonist), *N*-[(1*S*)-1-[[4-[(2*S*)-2-[[(2,4-dichlorophenyl)sulfonyl]amino]-3-hydroxy-1-oxopropyl]-1-piperazinyl]carbonyl]-3-methylbutyl]benzo[b]thiophene-2-carboxamide (GSK1016790A: selective TRPV4 agonist), and cis-4-phenyl-2-propionamidotetralin (4PPDOT, a selective MT_2_R antagonist) were purchased from Tocris Bioscience (Bristol, UK). *N*-[2-(2-methoxy-6H-isoindolo[2,1-a]indol-11-yl)ethyl]butanamide (IIK7, a selective MT_2_ receptor agonist [48]) was purchased from Sigma-Aldrich (St. Louis, MO, USA).

### 2.2. Cell Culture and Transient Transfection

A human non-pigmented ciliary epithelial (59HCE) immortalized cell line was supplied by Dr. Coca-Prados (Yale University). Cells were grown in high glucose Dulbecco’s modified Eagle’s medium (Gibco/Invitrogen, Carlsbad, CA) containing 10% fetal bovine serum (Sigma-Aldrich, St. Louis, MO, USA) and 0.05 mg/mL penicillin/streptomycin (Gibco/Invitrogen) at 37 °C in a humidified atmosphere of 5% CO_2_. After the culture reached the confluence, the cells were detached with 0.25% trypsin and seeded into 6 well plates or glass coverslips. All the experiments were performed using cells under 10–15 passages to assure assay reproducibility. Then, 59HCE cells were treated with a selective TRPV4 agonist, GSK1016790A (1–10,000 nM range), over 18 h, for the performance of the proximity ligation assay.

HEK-293T cells grown in DMEM (Gibco/Invitrogen, Carlsbad, CA, USA) were processed as previously described in References [49,50]. Briefly, HEK-293T cells growing in 6-well dishes were transfected transiently with the corresponding cDNA using the PEI (PolyEthylenImine, Sigma-Aldrich, St. Louis, MO, USA) method. The cells were incubated (for 4h) with the corresponding cDNA together with PEI (5.47 mM in nitrogen residues) and 150 mM NaCl in a serum-starved medium. After 4 h, the medium was replaced by a fresh complete culture medium.

### 2.3. Fusion Proteins and Expression Vectors

The human cDNAs for the D_3_, MT_1_, MT_2_, and GHS-R1a receptors cloned in pcDNA3.1 were amplified without their stop codons using sense and antisense primers. The primers harbored either unique BamHI and HindIII sites for D_3_; EcoRI and KpnI sites for GHS-R1a or Hind III; and BamH1 sites for MT_1_ and MT_2_. The fragments were subcloned to be in frame with an enhanced yellow fluorescent protein (pEYFP-N1; Clontech, Heidelberg, Germany) or an Rluc (pRluc-N1; PerkinElmer, Wellesley, MA) on the C-terminal end of the receptor to produce D_3_-RLuc, MT_1_–YFP, MT_2_–YFP and GHS-R1a-YFP fusion proteins.

### 2.4. Bioluminescence Resonance Energy Transfer (BRET) Assays

HEK-293T cells growing in 6-well plates were transiently co-transfected with a constant amount of cDNA encoding for D_3_R fused to Renilla luciferase (D_3_-Rluc) and with increasing amounts of cDNAs corresponding to MT_1_R, MT_2_R or ghrelin receptor GHS-R1a fused to the yellow fluorescent protein (MT_1_-YFP, MT_2_-YFP, GHS-R1a-YFP). 48 h post-transfection cells were washed twice in quick succession in HBSS (137 mM NaCl; 5 mM KCl; 0.34 mM Na_2_HPO4; 0.44 mM KH_2_PO_4_; 1.26 mM CaCl_2_; 0.4 mM MgSO_4_; 0.5 mM MgCl_2_ and 10 mM HEPES, pH 7.4) supplemented with 0.1% glucose (w/v), detached by gently pipetting and resuspended in the same buffer. To assess the number of cells per plate, we determined protein concentration using a Bradford assay kit (Bio-Rad, Munich, Germany) with bovine serum albumin dilutions as standards. To quantify YFP-fluorescence expression, we distributed the cells (20 μg protein) in 96-well microplates (black plates with a transparent bottom; Porvair, Leatherhead, UK). Fluorescence was read using a Mithras LB 940 (Berthold, Bad Wildbad, Germany) equipped with a high-energy xenon flash lamp, using a 10-nm bandwidth excitation and emission filters at 485 and 530 nm, respectively. YFP-fluorescence expression was determined as the fluorescence of the sample minus the fluorescence of cells expressing protein-Rluc alone. For the BRET measurements, the equivalent of 20 μg of cell suspension was distributed in 96-well microplates (white plates; Porvair), and we added 5 μM coelenterazine H (PJK GMBH, Kleinblittersdorf, Germany). Then, 1 min after coelenterazine H addition, the readings were collected using a Mithras LB 940 (Berthold, Bad Wildbad, Germany), which allowed the integration of the signals detected in the short-wavelength filter at 485 nm (440–500 nm) and the long-wavelength filter at 530 nm (510–590 nm). To quantify receptor-Rluc expression, we performed luminescence readings 10 min after addition of 5 μM coelenterazine H. The net BRET is defined as [(long-wavelength emission)/(short-wavelength emission)]-Cf where Cf corresponds to [(long-wavelength emission)/(short-wavelength emission)] for the Rluc construct expressed alone in the same experiment. The BRET curves were fitted assuming a single phase by a non-linear regression equation using the GraphPad Prism software (San Diego, CA, USA). BRET values are given as milli BRET units (mBU: 1000 × net BRET).

### 2.5. cAMP Level Determination

Two hours before initiating the experiment, 59HCE cells or HEK-293T cell-culture medium were exchanged to serum-starved DMEM medium. Then, the cells were detached, re-suspended in growing medium containing 50 µM zardaverine, and plated in 384-well microplates (2500 cells/well), pretreated (15 min) with the corresponding antagonists—or vehicle—and stimulated with agonists (15 min) before adding 0.5 μM forskolin or vehicle. Readings were performed after 1 h incubation at 25 °C. Homogeneous time-resolved fluorescence energy transfer (HTRF) measures were obtained using the Lance Ultra cAMP kit (PerkinElmer, Waltham, MA, USA) (see Reference [51]). Fluorescence at 665 nm was analyzed on a PHERAstar Flagship microplate reader equipped with an HTRF optical module (BMG Lab technologies, Offenburg, Germany).

### 2.6. Extracellular Signal-Regulated Kinase Phosphorylation Assays

To determine extracellular signal-regulated kinase 1/2 (ERK1/2) phosphorylation, 40,000 59HCE or HEK-293T cells/well were plated in transparent Deltalab 96-well plates and kept in the incubator for 48 h. Then, 2 to 4 h before the experiment the medium was replaced by serum-free medium. The cells were pre-treated at 25 °C for 10 min with antagonists or vehicle and stimulated for an additional 7 min with selective agonists. The cells were then washed twice with cold PBS before the addition of lysis buffer (a 20 min treatment). Afterward, 10 µL of each supernatant were placed in white ProxiPlate 384-well plates and ERK 1/2 phosphorylation was determined using an AlphaScreen^®^SureFire^®^ kit (Perkin Elmer), following the instructions of the supplier, and using an EnSpire^®^ Multimode Plate Reader (PerkinElmer, Waltham, MA, USA). The value of reference (100%) was the value achieved in the absence of any treatment (basal). The effect of ligands was given in percentage with respect to the basal value.

### 2.7. Label-Free Dynamic Mass Redistribution (DMR) Assay

Cell mass redistribution induced upon receptor activation was detected by illuminating the underside of a biosensor with polychromatic light. We then measured the changes in the wavelength of the reflected monochromatic light, which is a sensitive function of the index of refraction. The magnitude of the wavelength shift (in picometers) is directly proportional to the amount of mass redistribution. HEK-293T or 59HCE cells were seeded in 384-well sensor microplates to obtain 70–80% confluent monolayers constituted by approximately 10,000 cells per well. Previous to the assay, the cells were washed twice with assay buffer (HBSS with 20 mM HEPES, pH 7.15) and incubated for 2 h with assay-buffer containing 0.1% DMSO (24 °C, 30 µL/well). Hereafter, the sensor plate was scanned, and a baseline optical signature was recorded for 10 min before adding 10 µL of the selective antagonists for 30 min, followed by the addition of 10 µL of the selective agonists. All test compounds were dissolved in assay buffer. Then, DMR responses elicited by the agonists were monitored for at least 5000 s in an EnSpire^®^ Multimode Plate Reader (PerkinElmer, Waltham, MA, USA). The results were analyzed using EnSpire Workstation software v 4.10.

### 2.8. Human Eye Postmortem Samples

Donor human eyes were obtained from the Fundación Banco de Sangre y Tejidos de las Islas Baleares (Balearic Islands Blood and Tissue Bank). This biobank is supervised by the regional Balearic Islands Government, which is in charge of enforcing the principles in Helsinki declaration. All donations are made after appropriate filling and signing of informed consent by patients or their legal representatives. Two eyes of healthy normal subjects and two of glaucoma patients were used for the assays. The eyes were enucleated and collected without the cornea in sterile tubes and maintained in 4% paraformaldehyde in 0.1 M phosphate buffer (PB) (pH 7.2–7.4) at 4 °C until posterior processing. Eyes were dissected under a stereomicroscope (Zeiss), with a 0.8 mm curved tip forceps and sterile dissecting scissors, where we collected the iris and ciliary processes. Several washes in PBS were performed, and then, the specimens were cryoprotected in a sucrose gradient (from 11% to 33%) and embedded in tissue freezing medium (Tissue-Tek© OCT) until frozen with liquid N_2_. Vertical sections of control and glaucomatous human samples (10 µm thick) were collected using a cryostat (Leica, Nussloch, Germany) and were mounted from the same region. The samples were maintained at −20 °C until use.

### 2.9. Immunofluorescence Studies

HEK-293T cells were fixed in 4% paraformaldehyde for 15 min and then washed twice with PBS containing 20 mM glycine before permeabilization with the same buffer containing 0.2% Triton X-100 (5 min incubation). The cells were treated for 1 h with PBS containing 1% bovine serum albumin and labeled with a mouse anti-Rluc (1/100; MAB4400, Millipore) antibody and subsequently treated with Cy3-conjugated anti-mouse (1/200; 715-166-150; Jackson ImmunoResearch (red)) IgG secondary antibody (1 h each). Alternatively, cells were labeled with rabbit anti-D_3_R (#ab42114), anti-MT_1_R (#ab203038), or anti-MT_2_R (#ab115336) antibodies, all from Abcam (Cambridge, UK) and subsequently treated with Cy3-conjugated anti-rabbit (1/200; #711-166-152; Jackson ImmunoResearch (red)) IgG secondary antibody (1 h each). The samples were washed several times and mounted with 30% Mowiol (Calbiochem). Samples were observed under a Leica SP2 confocal microscope (Leica Microsystems).

### 2.10. In Situ and In Vitro Proximity Ligation Assay (PLA)

The proximity ligation assay (PLA) allows the detection of molecular interactions between two endogenous proteins ex vivo. PLA requires both receptors to be sufficiently close (<16 nm) to allow double-strand formation of the complementary DNA probes conjugated to the antibodies. Using the PLA, the heteromerization of D_3_R with MT_1_ and MT_2_ receptors was detected in non-pigmented epithelial ciliary body cells (59HCE) and, in situ, in the human ciliary body sections of healthy and glaucomatous donors.

The presence/absence of receptor–receptor molecular interactions in the samples was detected using the Duolink II In Situ PLA Detection Kit (developed by Olink Bioscience, Uppsala, Sweden; and now distributed by Sigma-Aldrich as Duolink^®^ using PLA^®^ Technology). The PLA probes were obtained after conjugation of the primary anti-D_3_R antibody (ab42114, Abcam, Cambridge, UK) to a PLUS oligonucleotide (DUO92009, Sigma-Aldrich, St. Louis, MO, USA), and the anti-MT_1_R (ab203038, Abcam, Cambridge, UK) and -MT_2_R (ab115336, Abcam, Cambridge, UK) antibodies to a MINUS oligonucleotide (DUO92010, Sigma-Aldrich, St. Louis, MO, USA). The specificity of antibodies was tested in non-transfected HEK-293T cells (see Appendix A Figure A3). Samples were fixed in 4% paraformaldehyde for 15 min and then washed twice with PBS containing 20 mM glycine before permeabilization with PBS-glycine containing 0.2% Triton X-100 (5 min incubation for the 59HCE cells and 15 min for the ciliary body sections). After permeabilization, the samples were washed in PBS at room temperature and incubated in a preheated humidity chamber for 1 h at 37 °C with the blocking solution provided in the PLA kit. Then, the samples were incubated overnight with the PLA probe-linked antibodies (1:100 dilution for all antibodies) at 4 °C. After washing, the samples were incubated with the ligation solution for 1 h, and then washed and subsequently incubated with the amplification solution for 100 min (both steps at 37 °C in a humid chamber). Nuclei were stained with Hoechst (1/100; Sigma-Aldrich, St. Louis, MO, USA). Mounting was performed using 30% Mowiol (Calbiochem). Negative controls were performed by omitting the anti-D_3_R-PLUS antibody. Samples were observed using a Leica SP2 confocal microscope (Leica Microsystems, Mannheim, Germany) equipped with an apochromatic 63× oil-immersion objective (N.A. 1.4), and with 405 nm and 561 nm laser lines. For each field of view, we acquired a stack of two channels (one per staining) and 5 Z planes with a step size of 1 µm. The ratio r (number of red spots/cell) was determined on the maximum projection of each image stack using the Duolink Image tool software.

### 2.11. Data Analysis

Data, expressed as the mean ± SEM, were obtained from at least five independent experiments. Data comparisons were analyzed by one-way ANOVA or two-way ANOVA, followed by Bonferroni’s post-hoc test. The normality of populations and homogeneity of variances were tested before the ANOVA. Statistical analysis was undertaken only when each group size was at least n = 5, n being the number of independent variables (technical replicates were not treated as independent variables). Differences were considered significant when *P* ≤ 0.05. Statistical analyses were carried out with GraphPad Prism software version 5 (San Diego, CA, USA). Outliers tests were not used, and all data points (mean of replicates) were used for the analyses.

## 3. Results

### 3.1. Dopamine D_3_ Receptors Interact with Melatonin MT_1_ Receptors in the HEK-293T Cells

**Figure 1:**
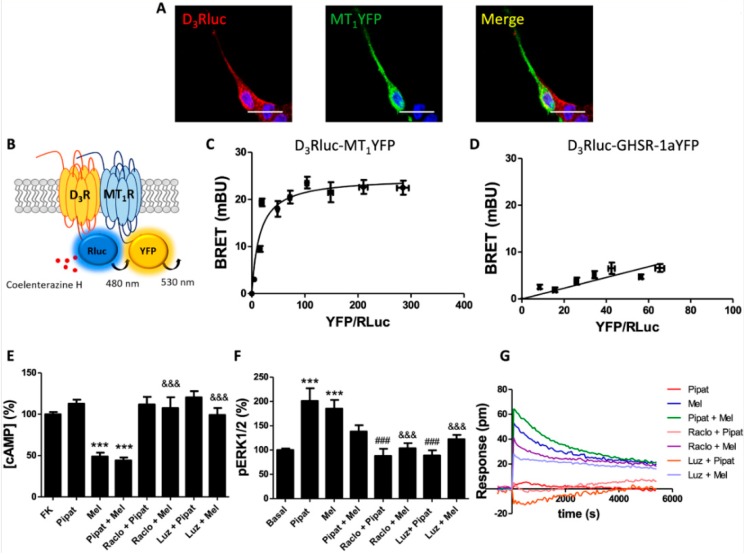
Molecular interaction between D_3_ and MT_1_ receptors, and heteromer-mediated signaling. (**A**) Confocal microscopy images of HEK-293T cells co-expressing D_3_R-Rluc (2 µg) and MT_1_R-YFP (2 µg). D_3_ receptor (red) was identified by immunocytochemistry using anti-Rluc antibodies. MT_1_ receptor (green) was identified from the fluorescence of YFP-containing fusion proteins. Co-localization is shown in the panel on the right (yellow). Cell nuclei were stained with Hoechst (blue channel). Scale bar: 20 μm. (**B**) Scheme of the bioluminescence resonance energy transfer (BRET) assay. (**C**,**D**) BRET saturation experiments performed using HEK-293T cells co-transfected with D_3_R-Rluc cDNA (0.7 μg) and increasing amounts of MT_1_R-YFP cDNA (0–1.4 μg cDNA) (**C**) or GHSR-1a-YFP cDNA (0–2.5 μg cDNA) as a negative control (**D**). BRET data are expressed as the mean ± S.D. of 8 different experiments performed in duplicates. mBU: milliBret units. HEK-293T cells transfected with cDNA encoding for D_3_R (1 μg) and MT_1_R (1 μg) were pre-treated or not with receptor antagonists (1 μM raclopride for D_3_R or 1 μM luzindole for MT_1_R) and then subsequently treated with agonists (100 nM 7-OH-PIPAT for D_3_R or 1 μM melatonin for MT_1_R), alone or in combination. (**E**) cAMP data were expressed as a % over 0.5 μM forskolin-induced levels. (**F**) ERK1/2 phosphorylation was analyzed using an AlphaScreen^®^SureFire^®^ kit (Perkin Elmer). ERK1/2 phosphorylation data are expressed as % with respect to basal levels. In cAMP accumulation and MAPK activation assays, values are the mean ± S.E.M. of 6 different experiments performed in triplicates. One-way ANOVA followed by Bonferroni’s multiple comparison post-hoc tests were used for statistical analysis. (*** *p* < 0.001; versus treatment with forskolin in cAMP or basal in pERK assays). (### *p* < 0.001; versus treatment with 7-OH-PIPAT alone). (&&& *p* < 0.001; versus treatment with melatonin alone). (**G**) label-free dynamic mass redistribution (DMR) tracings are representing the picometer-shifts of reflected light wavelengths over time upon ligand treatment.

**Figure A1:**
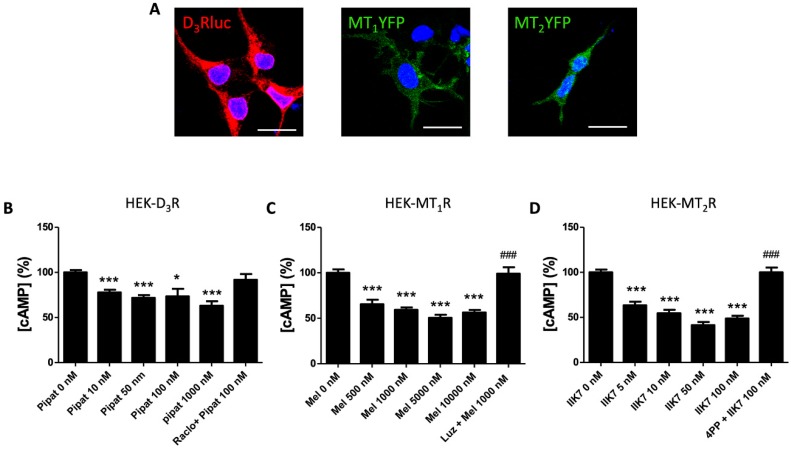
Membrane receptor expression and cAMP responses in single-transfected HEK-293T cells. (**A**) Confocal microscopy images of HEK-293T cells transfected with cDNAs for D_3_R-Rluc (2 μg), MT_1_R-YFP (2 μg), or MT_2_R-YFP (2 μg). D_3_ receptors (red) were identified by immunocytochemistry using anti-Rluc antibodies. MT_1_ and MT_2_ receptors (green) were identified from the fluorescence of YFP-containing fusion proteins. Cell nuclei were stained with Hoechst (blue channel). Scale bar: 20 μm. Panels (**B**–**D**) HEK-293T cells transfected with cDNAs encoding for D_3_R (1.5 μg) (**B**), MT_1_R (1.5 μg) (**C**) or MT_2_R (1.5 μg) (**D**) cells were pre-treated with vehicle or antagonists (1 μM raclopride for D_3_R, 1 μM luzindole for MT_1_R or 1 μM 4PPDOT for MT_2_R), and then subsequently treated with different concentrations of selective agonists (10–1000 nM 7-OH-PIPAT for D_3_R, 0.5–10 μM melatonin for MT_1_R or 5–100 nM IIK7 for MT_2_R). cAMP accumulation was detected by TR-FRET in the presence of 0.5 μM forskolin. cAMP production is expressed as % of levels obtained by 0.5 μM forskolin (0 nM agonist). Values are the mean ± S.E.M. of 6 different experiments performed in triplicates, and one-way ANOVA followed by Bonferroni’s multiple comparison post-hoc tests were used for statistical analysis. (* *p* < 0.05, *** *p* < 0.001; versus treatment with forskolin). (### *p* < 0.001; versus treatment with 1 μM melatonin or 100 nM IIK7 alone).

To determine whether the dopamine D_3_ receptor (D_3_R) could interact with the melatonin MT_1_ receptor, we first performed immunocytochemical assays in a heterologous expression system. HEK-293T cells were transfected with cDNAs coding for D_3_R-Rluc or MT_1_R-YFP. Expression of D_3_R-Rluc was detected using an anti-Rluc primary antibody followed by a secondary Cy3-conjugated antibody, and expression of MT_1_R-YFP was detected using the YFP’s fluorescence. Receptor expression was found in different cell compartments, including the plasma membrane (Appendix A Figure A1A, left and center panels). When the cells were co-expressing D_3_R-Rluc and MT_1_R-YFP, a significant degree of co-localization was observed (yellow in Figure 1A).

As co-localization is not direct evidence of interaction, we used an energy-transfer biophysical approach aimed at identifying direct physical interactions. Bioluminescence resonance energy transfer (BRET) assays were performed in HEK-293T cells expressing a constant amount of D_3_R-Rluc and increasing amounts of MT_1_R-YFP (Figure 1B). A saturation BRET curve was obtained (BRET_max_ 24.6 mBU, BRET_50_ 14.9) (Figure 1C), indicating that D_3_R and MT_1_R physically interacted. As a negative control, D_3_R was co-expressed with a non-interacting partner, the ghrelin receptor GHSR-1a. In this case, the cells were co-transfected with a constant amount of D_3_R-Rluc and increasing amounts of GHSR-1aYFP. A linear signal was obtained, indicating the lack of interaction between these receptors (Figure 1D). These results indicated that D_3_ and MT_1_ may form heteroreceptor complexes in a heterologous expression system.

### 3.2. Functional Characterization of D_3_-MT_1_ Heteroreceptor Complexes in HEK-293T Cells

**Figure A2:**
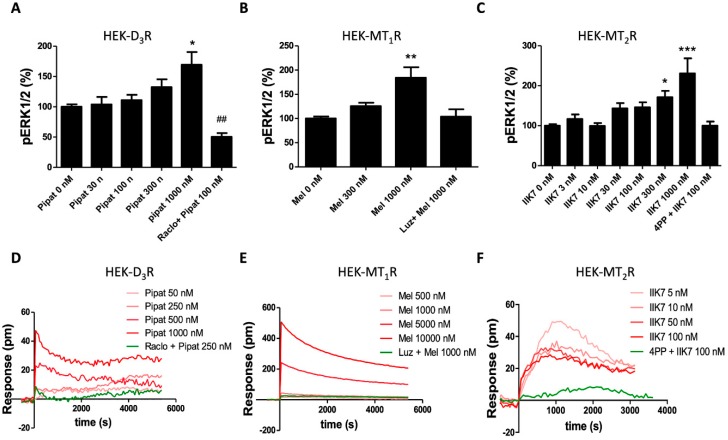
ERK phosphorylation and DMR responses in single-transfected HEK-293T cells. HEK-293T cells transfected with cDNA encoding for D_3_R (1.5 μg) (**A**,**D**), MT_1_R (1.5 μg) (**B**,**E**), or MT_2_R (1.5 μg) (**C**,**F**) were pre-treated with vehicle or antagonists (1 μM raclopride for D_3_R, 1 μM luzindole for MT_1_R, or 1 μM 4PPDOT for MT_2_R), and then subsequently treated with different concentrations of agonists (30–1000 nM 7-OH-PIPAT for D_3_R, 0.3–10 μM melatonin for MT_1_R, or 3–1000 nM IIK7 for MT_2_R). ERK1/2 phosphorylation (**A**–**C**) was analyzed using an AlphaScreen^®^SureFire^®^ kit (Perkin Elmer). ERK1/2 phosphorylation data are expressed as % with respect to basal levels (0 nM agonist). Values are the mean ± S.E.M. of seven different experiments performed in triplicates, and one-way ANOVA followed by Bonferroni’s multiple comparison post-hoc tests were used for statistical analysis. (* *p* < 0.05, ** *p* < 0.01, *** *p* < 0.001; versus basal). (## *p* < 0.001; versus treatment with 100 nM 7-OH-PIPAT alone). (**D**–**F**) DMR tracings representing the picometer-shifts of reflected light wavelengths over time upon agonist ligand treatment.

Before estimating whether D_3_R and MT_1_R signaling properties were altered by D_3_R–MT_1_R heteromerization, we performed assays in single-transfected cells. These preliminary assays consisted of dose-response curves, to select the single agonist concentration for use in further assays performed in cells expressing the two receptors. Cognate heterotrimeric G proteins are Gi/o for melatonin and D_3_ receptors [26]. Thus, activation of these receptors leads to a decrease in cytosolic cAMP levels. We have confirmed that this is the case in single transfected cells (Appendix A Figure A1B,C; see also our recent report [52]), thereby reproducing data reported in References [34,53]. HEK-293T cells expressing D_3_R or MT_1_ and incubated with the selective agonists, 7-OH-PIPAT for D_3_ and melatonin for MT_1_, led to significant decreases in the forskolin-induced cytosolic cAMP levels. The blockade was afforded by the respective antagonist, raclopride, or luzindole (Appendix A Figure A1B,C). Next, we analyzed the mitogen-activated protein kinase (MAPK) signaling pathway, which may be mediated by G-protein-dependent or independent mechanisms. Experiments performed in single-transfected cells HEK-293T cells expressing D_3_R or MT_1_R led to a dose-response increase in phosphorylated ERK1/2 levels, which was reversed by the respective antagonists (Appendix A Figure A2A,B). Finally, we used a label-free approach based on the cell’s dynamic mass redistribution (DMR). Increasing agonist concentrations were assayed in single-transfected HEK-293T cells expressing D_3_R or MT_1_R treated with 7-OH-PIPAT or with melatonin, respectively. In both cases, we could observe significant DMR outputs that were reverted by the respective antagonists (Appendix A Figure A2D,E).

Next, HEK-293T cells were transfected with both D_3_R and MT_1_R and then treated with 100 nM 7-OH-PIPAT and 1 µM melatonin, alone or in combination. Melatonin induced a decrease in the forskolin-induced cAMP levels to a similar extent to that produced in cells only expressing MT_1_R. In contrast, 7-OH-PIPAT did not produce any effect (Figure 1E). These results suggest that the co-expression of MT_1_R blocks the 7-OH-PIPAT-induced D_3_R activation and Gi-mediated signaling. The effect in simultaneous treatment with agonists was similar to that produced by melatonin alone. When the cells were pre-treated with antagonists, we could observe that the melatonin effect was not only abolished by the MT_1_R antagonist, luzindole, but also by the D_3_R antagonist, raclopride (Figure 1E). This cross-antagonism, consisting of the antagonist of one receptor blocking the signal of the partner receptor, is often found in GPCR heteroreceptor complexes.

When the MAPK signaling pathway was analyzed in the HEK-293T cells co-expressing D_3_ and MT_1_ receptors, a significant ERK1/2 phosphorylation was observed when the cells were treated with 7-OH-PIPAT or melatonin. These results showed that MT_1_R did not block 7-OH-PIPAT-induced D_3_R signaling in the MAPK pathway (Figure 1F). Interestingly, the effect was much lower when the cells were stimulated simultaneously with both agonists. Simultaneous co-activation resulting in reduced signaling is known as negative cross-talk and has been reported for other GPCR heteromers. When cells were pre-treated with antagonists, a bi-directional cross-antagonism was detected, which consisted of the antagonists of either receptor blocking the activation of the partner receptor in the heteromer. Indeed, the D_3_R antagonist raclopride inhibited not only the effect of 7-OH-PIPAT but also the effect of melatonin, and the MT_1_R antagonist luzindole inhibited the effect of each agonist (Figure 1F).

DMR assays performed in the cells co-expressing D_3_R and MT_1_R led to results that were similar to those observed in [cAMP] determination assays. While melatonin produced a significant effect on DMR, 7-OH-PIPAT did not produce any effect (Figure 1G). When the cells were pre-treated with antagonists, we observed a cross-antagonism phenomenon, as the melatonin effect was not only inhibited by luzindole, but also by raclopride.

Together, the results showed that the consequences of D_3_-MT_1_ heteroreceptor complex formation included a blockade of D_3_R-Gi coupling, a negative cross-talk in signaling towards the MAPK pathway, and a cross-antagonism.

### 3.3. Dopamine D_3_ Receptors Interact with Melatonin MT_2_ Receptors in HEK-293T Cells

**Figure 2:**
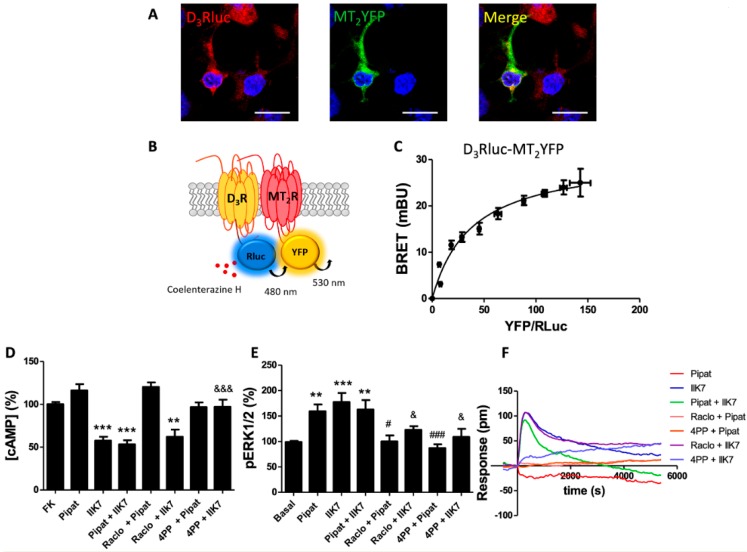
Molecular interaction between D_3_ and MT_2_ receptors, and heteromer-mediated signaling. (**A**) Confocal microscopy images of HEK-293T cells co-expressing D_3_R-Rluc (2 µg) and MT_2_R-YFP (2 µg). D_3_ receptor (red) was identified by immunocytochemistry using anti-Rluc antibodies. MT_2_ receptor (green) was identified from the fluorescence of YFP-containing fusion proteins. Co-localization is shown in the panel on the right (yellow). Cell nuclei were stained with Hoechst (blue channel). Scale bar: 20 μm. (**B**) Scheme of the BRET assay. (**C**) BRET saturation experiments performed using HEK-293T cells co-transfected with D_3_R-Rluc cDNA (0.7 μg) and increasing amounts of MT_2_R-YFP cDNA (0–2.3 μg cDNA). BRET data are expressed as the mean ± S.D. of 8 different experiments performed in duplicates. mBU: milliBret units. Panels (**D**–**F**) Signaling in HEK-293T cells transfected with cDNA encoding for D_3_R (1 μg) and MT_2_R (1 μg) were pre-treated with antagonists (1 μM raclopride for D_3_R or 1 μM 4PPDOT for MT_2_R) or vehicle, and then subsequently treated with selective agonists (100 nM 7-OH-PIPAT for D_3_R and 300 nM IIK7 for MT_2_R), individually or in combination. (**D**) [cAMP] (expressed as % with respect to treatment with 0.5 μM forskolin) was detected by TR-FRET. (**E**) ERK1/2 phosphorylation was analyzed using an AlphaScreen^®^SureFire^®^ kit (Perkin Elmer). ERK1/2 phosphorylation data are expressed as % with respect to basal levels. In cAMP accumulation and MAPK signaling assays, the values are the mean ± S.E.M. of 6 different experiments performed in triplicates, and one-way ANOVA followed by Bonferroni’s multiple comparison post-hoc tests were used for statistical analysis. (** *p* < 0.01, *** *p* < 0.001; versus treatment with forskolin in cAMP or basal in pERK assays). (# *p* < 0.05, ### *p* < 0.001; versus treatment with 7-OH-PIPAT alone). (& *p* < 0.05, &&& *p* < 0.001; versus treatment with IIK7 alone). (**F**) DMR tracings representing the picometer-shifts of reflected light wavelengths over time upon ligand treatment.

We considered the possibility of the D_3_R also interacting with the MT_2_R. Accordingly, we followed the same strategy described earlier for MT_1_R. Immunocytochemical assays showed the presence of the receptor in different cell compartments, including the cell membrane (Appendix A Figure A1A, right). In double-transfected cells, a high degree of co-localization between D_3_R and MT_2_R was observed (Figure 2A). Next, the BRET assay, schematized in Figure 2B, undoubtedly proved that D_3_R might form heteroreceptor complexes with MT_2_R in a heterologous expression system. The saturation curve was characterized by a BRET_max_ of 30.7 mBU and a BRET_50_ of 38.2.

### 3.4. Functional Characterization of the D_3_-MT_2_ Heteroreceptor Complexes in HEK-293T Cells

Dose-response assays in HEK-293T cells expressing MT_2_R were performed using a selective MT_2_R agonist, IIK7. The results were consistent with Gi coupling, with MAPK activation, and with marked DMR readings. Pretreatment with the MT_2_R antagonist 4P-PDOT blocked the MT_2_R agonist-induced effect (Appendix A Figure A1D and Figure A2C,F).

In forskolin-pretreated HEK-293T cells co-expressing D_3_R and MT_2_R, treatment with 7-OH-PIPAT did not decrease cAMP levels, while IIK7 was able to produce a signal similar to that produced in single-transfected cells. This result indicated that co-expression of MT_2_R blocks 7-OH-PIPAT-induced Gi-mediated signaling (Figure 2D), in full resemblance to what was observed for the D_3_R–MT_1_R heteromer. However, for the D_3_R-MT_2_R heteromer, no cross-antagonism was detected. When activation of the MAPK pathway was analyzed in cotransfected HEK-293T cells, we observed the same phenomena encountered for the D_3_R–MT_1_R heteromer. Both agonists were able to elicit a signal when used separately but a negative cross-talk was detected in simultaneous co-activation. In this experiment, the bi-directional cross-antagonism effect was observed, as both raclopride and 4P-PDOT were able to block the signal elicited by either D_3_R or MT_2_R activation (Figure 2E). Finally, when the DMR responses were analyzed, 7-OH-PIPAT did not produce any signal, while IIK7 produced a significant effect. Again, as in the cAMP assays, no cross-antagonism was detected. The IIK7-induced signal was only blocked by MT_2_R antagonist 4P-PDOT and not by the D_3_R antagonist raclopride. To sum up, the results showed that the consequences of D_3_-MT_2_ heteroreceptor complex formation included a blockade of D_3_R-Gi coupling and a negative cross-talk plus bi-directional cross-antagonism when signaling towards the MAPK pathway was analyzed.

### 3.5. Detection and Functional Characterization of D_3_-MT_1_ and D_3_-MT_2_ Heteroreceptor Complexes in Human Non-Pigmented Ciliary Body Epithelial Cells

**Figure 3:**
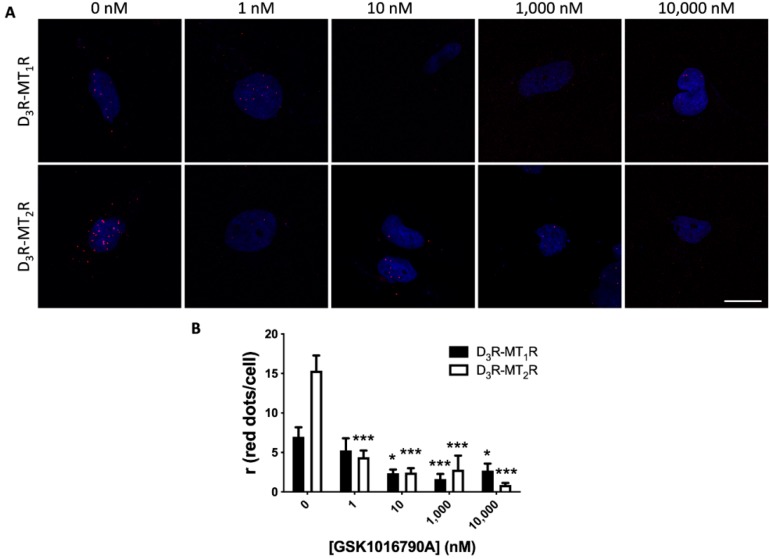
D_3_-MT_1_ and D_3_-MT_2_ heteroreceptor expression in human 59HCE cells. Proximity ligation assay (PLA) was performed as described in Methods to detect D_3_-MT_1_ and D_3_-MT_2_ receptor complexes in the 59HCE non-pigmented ciliary body epithelial cell line, using specific antibodies against D_3_ and either MT_1_ or MT_2_ receptors. (**A**) Cells were treated with different concentrations of GSK1016790A, a TRPV4 agonist. Representative images corresponding to stacks of 5 sequential planes are shown. Cell nuclei were stained with Hoechst (blue) and heteroreceptor clusters appear as red dots. (**B**) Ratio (r; number of red spots/cell-containing spots) is the mean ± S.E.M. of counts in 5 different fields from every sample (n = 5). One-way ANOVA followed by Bonferroni’s multiple comparison post-hoc test were used for statistical analysis. (* *p* < 0.05, *** *p* < 0.001; versus 0 nM GSK1016790A). Scale bar: 20 μm.

**Figure A3:**
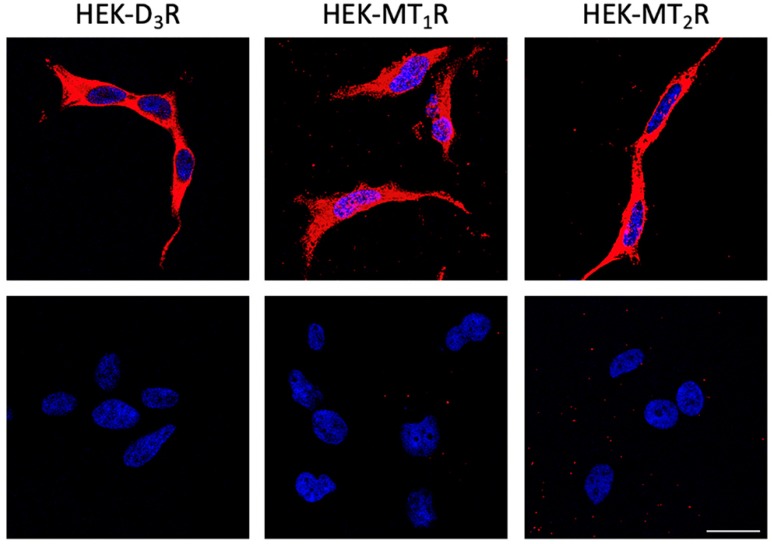
The specificity of antibodies against D_3_, MT_1_, and MT_2_ receptors. Confocal microscopy images of the HEK-293T cells untransfected (bottom) or transfected with cDNAs coding for D_3_, MT_1_, or MT_2_ receptors (0.5 μg). The cells were treated with primary antibodies against either D_3_, MT_1_, or MT_2_ receptors (as indicated), and subsequently treated with a Cy3 secondary antibody (red). Images were taken near the bottom of the cell and, therefore, reflect the labeling in the membrane in contact with the slide. Cell nuclei were stained with Hoechst (blue channel). Scale bar: 20 μm.

To determine whether D_3_R–MT_1_R and D_3_R–MT_2_R heteromers occur in natural sources, we took advantage of a technique that allows the detection of clusters of two receptors in fixed cells or tissue sections, i.e., the in situ proximity ligation assay (PLA). The PLA was performed in the non-pigmented human ciliary body epithelial 59HCE cell line, which expresses three receptors. Specific antibodies against D_3_, MT_1_, and MT_2_ receptors were used (see Appendix A Figure A3) and punctuated red marks were visualized surrounding stained nuclei. This outcome demonstrated the existence of D_3_-MT_1_ and D_3_-MT_2_ receptor complexes/clusters (Figure 3A, top and bottom left—0 nM).

**Figure 4:**
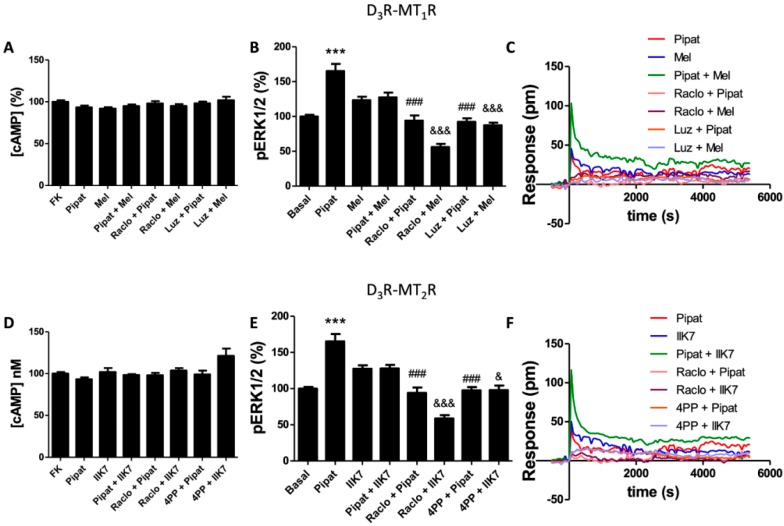
Effect of dopamine and melatonin receptor agonists in human 59HCE cells. Signaling was assayed in the 59HCE cells pre-treated with antagonists (1 μM raclopride for D_3_R, 1 μM luzindole for MT_1_R, or 1 μM 4PPDOT for MT_2_R) or vehicle, and then subsequently treated with agonists (100 nM 7-OH-PIPAT for D_3_R and either 1 μM melatonin for MT_1_R (**A**–**C**) or 300 nM IIK7 for MT_2_R (**D**–**F**)), individually or in combination. (**A**,**D**) cAMP production is expressed as % over 0.5 μM forskolin-induced [cAMP] increases. (**B**,**E**) ERK1/2 phosphorylation data are expressed as % with respect to basal levels. In cAMP accumulation and MAPK signaling assays, the values are the mean ± S.E.M. of 5 different experiments performed in triplicates, and one-way ANOVA followed by Bonferroni’s multiple comparison post-hoc tests were used for statistical analysis. (*** *p* < 0.001; versus basal in pERK assays). (### *p* < 0.001; versus treatment with 7-OH-PIPAT alone). (& *p* < 0.05, &&& *p* < 0.001; versus treatment with melatonin or IIK7 alone). (**C**,**F**) DMR tracings representing the picometer-shifts of reflected light wavelengths over time upon ligand treatment. Note that data related to D_3_R agonists are the same in the upper and lower panels; for clarity, we decided to keep six panels instead of including all the information in three panels.

Next, we studied the signaling properties of D_3_-MT_1_ and D_3_-MT_2_ heteroreceptor complexes in the 59HCE cells using the approaches described in previous subsections. When cytosolic cAMP levels were analyzed in the cells treated with 0.5 µM forskolin, the D_3_R agonist 7-OH-PIPAT, in agreement with the heteromer’s print identified in the HEK-293T cells, did not produce any effect (Figure 4A,D).

Interestingly, and as previously described by Alkozi et al., [34], none of the melatonin receptor agonists, melatonin or IIK7, were able to decrease cAMP levels in these cells. Next, we analyzed ERK1/2 phosphorylation, which was significantly enhanced when 7-OH-PIPAT was added (Figure 4B,E). When the cells were treated with 7-OH-PIPAT in combination with melatonin or with IIK7, a negative cross-talk was observed, whereas a bi-directional cross antagonism occurred for both the D_3_R–MT_1_R and D_3_R–MT_2_R heteromers. Finally, interpretation of the DMR data resulting from activation of the receptors in the two heteromers was complex (Figure 4C,F). Perhaps the most relevant finding was the bi-directional cross-antagonism detected for both D_3_R–MT_1_R and D_3_R–MT_2_R heteromers expressed in the 59HCE cells. Apart from the evidence on heteromer formation, the results showed that neither D_3_, MT_1_, or MT_2_ receptors were coupled functionally to Gi and that D_3_R–MT_1_R and D_3_R–MT_2_R signaling was biased towards the MAPK pathway.

### 3.6. Differential Expression of D_3_-MT_1_ and D_3_-MT_2_ Heteroreceptor Complexes in the Glaucomatous Eye

We wondered whether intraocular hypertension correlated with the altered expression of dopamine and melatonin heteroreceptor complexes. First of all, we took advantage of an in vitro model of hypertension, described in Reference [54], consisting of the activation of the transient receptor potential vanilloid 4 (TRPV4) channel. Such activation in 59HCE cells using GSK1016790A mimics the ion fluxes that drive the increase in hydrostatic pressure that occurs in the hypertensive/glaucomatous eye. TRPV4 stimulation by application of the agonist, GSK1016790A, was followed by PLA to detect the D_3_R-containing heteromers. Remarkably, increasing GSK1016790A concentrations translated into a significant decrease in the PLA signal for D_3_-MT_1_ and D_3_-MT_2_ heteromers, and the effect was dose-dependent. The reduction was up to 45% for D_3_R–MT_1_R heteromer expression, and up to 95% in the case of D_3_R–MT_2_R heteromers (Figure 3A,B). Therefore, we could conclude that mimicking glaucomatous conditions in a cell model reduces the expression of D_3_-MT_1_ and D_3_-MT_2_ heteroreceptor complexes.

**Figure 5:**
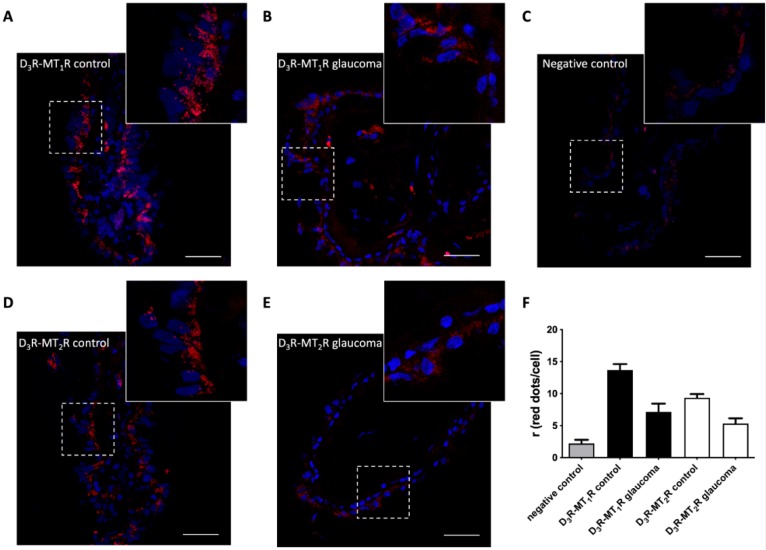
D_3_-MT_1_ and D_3_-MT_2_ receptor heteromer expression in the human ciliary body. In situ proximity ligation assays were performed as described in Materials and Methods using human ciliary body sections from age-matched intraocular pressure (IOP) normotensive individuals (control) (**A**,**C**,**D**) and glaucoma patients (**B**,**E**). Confocal microscopy images (superimposed sections) are shown. D_3_-MT_1_ (**A**,**B**) and D_3_-MT_2_ (**D**,**E**) receptor heteromers appear as red clusters and Hoechst-stained nuclei appear in blue. (**C**) A negative control obtained by omitting one of the primary antibodies is shown. The ratio (number of red spots/cell) for the indicated samples is shown in (**F**). Data are the mean ± S.E.M. of counts in 6 different fields (each image covered an area of 238 μm^2^) from every sample from the controls (n = 2) or glaucoma patients (n = 2). Scale bar: 40 μm.

Finally, exploratory experiments on heteromer expression were performed in the postmortem samples obtained from glaucoma patients and age-matched controls. Given the limited availability of these unique samples, which in part reflects ethical issues, we had access to two control (healthy) and two glaucomatous eyes. Accordingly, statistical analysis could not be performed on the results described below. PLA was performed in ciliary body sections of glaucoma patients and age-matched controls. In the samples from normotensive eyes, marked expression of D_3_R–MT_1_R and D_3_R–MT_2_R heteromers was found in the basal membrane of the non-pigmented epithelium. No heteromer expression was found in the stroma of the ciliary processes (Figure 5A,D). A negligible PLA signal was found in the negative controls performed by omitting one of the primary antibodies (Figure 5C). When samples from glaucoma cases were analyzed, we observed a significant reduction in the number dots for both heteromers (Figure 5B,E), with up to a 50% reduction in D_3_R–MT_1_R heteromer expression and a reduction of up to 45% in the case of D_3_R–MT_2_R heteromers (Figure 5F). Together with the results obtained in the 59HCE cells, these data point to a reduction of dopamine–melatonin heteroreceptor complexes under conditions of elevated intraocular pressure.

## 4. Discussion

Dopamine, one of the primary neurotransmitters of the central nervous system, participates in almost any higher-order function, from motor control to emotion control, and from cognition to behavior. Dopamine is also a neurohormone acting in the periphery via the same receptors that are expressed in the brain. The difference is that the relevant actions of dopamine are mainly exerted by neuronal receptors, while in the periphery, these receptors are expressed in almost any kind of cell type. A prospection study, made in our laboratory, showed the expression of the D_3_R in different cells of the eye structure, including those that controlled IOP. These expression results fit with pharmacological studies showing that different D_3_R agonists may lower IOP and that the effect is lost in D_3_R^-/-^ mice [43,44,45]. From earlier studies by the laboratory of S. George [55,56,57] it is known that dopamine receptors may directly interact with themselves to form homodimers or with other GPCRs. In a previous study, we also discovered D_1_-D_3_ heteroreceptor complexes with high relevance in Parkinson’s disease-associated dyskinesia [53]. For these reasons, we explored whether the D_3_R expressed in ocular structures could form functional heteromers with other GPCR of significant relevance for eye physiology.

Melatonin is an example of a neurohormone produced by the pineal gland that may also be produced (and released) elsewhere. Enzymes that participate in the synthesis of melatonin are expressed in different cell types and the indoleamine participates in IOP regulation. Although the retina was among the first identified structures able to synthesize melatonin [58,59], other eye cell types express melatonin synthesizing enzymes (see 1. Introduction).

Our results decipher one of the mechanisms underlying the dopamine–melatonin interactions described to occur in the eye. Based on prototypic heteromer prints that occur similarly in heterologous expression systems and natural cell lines, and by further confirmation in natural sources by PLA, we discovered novel physiologically relevant receptor–receptor interactions. The D_3_R, expressed in ciliary body epithelial cells, may interact with either MT_1_R or MT_2_R. Canonical coupling of melatonin receptors individually expressed in a heterologous system is to Gi (see References [25,52] and references therein). Dopamine receptors are either coupled to Gs (D_1_ and D_5_) or Gi (D_2_, D_3_ and D_4_). Remarkably, in the D_3_R–MT_1_R and the D_3_R–MT_2_R heteromer, the dopamine receptor did not couple to Gi but was still functional, as dopamine receptor agonists could activate the MAPK pathway. Indeed, heteromerization produced a biased functionality, i.e., a given agonist of the D_3_R, e.g., 7-OH-PIPAT may engage Gi-mediated signaling but only in the right context [60]. Our results prove antagonistic interactions, thus constituting a base to understand why the levels of dopamine and melatonin in the eye are oppositely varying according to the 24-h circadian rhythm [39,40]. Not only the presence of melatonin receptors and heteromer formation alters D_3_R-mediated signaling, but coactivation of receptors—in heteromers—by moderate levels of the indoleamine and of dopamine would result in a decrease in the overall signal transduction output. Accordingly, these heteromers mediate dopamine/melatonin antagonistic actions in the eye.

Considering IOP regulation by melatonin, circadian rhythms, and opposite melatonin/dopamine circadian levels in the eye; the results of heteromer expression in both a cell model of eye-related hypertension, namely 59HCE cells treated with a TRPV4 agonist, and the human eye (from IOP normotensive and hypertensive individuals) were relevant. They provided evidence of a correlation between heteromer D_3_R–MT_1_R and D_3_R–MT_2_R complex disruption and elevated IOP. Despite the few studies addressing GPCR heteromer expression in disease, often the results are quite relevant. On the one hand, they provide the basis for confirming whether the heteromer is expressed or not and, accordingly, whether the heteromer may be a therapeutic target. On the other hand, the differential functionality may lead to understanding of the consequences, at the signaling level, of the maintenance or the disassembly of heteroreceptor complexes. This will provide reliable information on the pathophysiology and factors affecting the course of the disease.

Based on our results, we think that future directions should include interventions assaying IOP after infusions in the eye of a combination of melatonin and either D_3_R agonists or antagonists at different times of the day in wild type animals and in a glaucoma animal model, such as the aged DBA/2J mice [61,62]. From the results of such analysis, a potential therapy would arise for the assessment of the hypertensive eye.
